# Extracellular resistance is sensitive to tissue sodium status; implications for bioimpedance-derived fluid volume parameters in chronic kidney disease

**DOI:** 10.1007/s40620-019-00620-3

**Published:** 2019-06-18

**Authors:** Nicos Mitsides, Damien McHugh, Agnieszka Swiecicka, Roshni Mitra, Paul Brenchley, Geoff J. M. Parker, Sandip Mitra

**Affiliations:** 1 Division of Cardiovascular Sciences, School of Medical Sciences, Faculty of Biology, Medicine and Health, The University of Manchester, Manchester Academic Health Science Centre, Manchester, UK; 2 Nephrology Department, Salford Royal Hospital NHS Foundation Trust, Stott Lane, Salford, M6 8HD UK; 3 NIHR Devices for Dignity Medical Technology Co-operative, Sheffield, UK; 4 Quantitative Biomedical Imaging Laboratory, Faculty of Biology, Medicine and Health, University of Manchester, Manchester Academic Health Science Centre, Manchester, UK; 5 Andrology Research Unit, Division of Gastroenterology, Endocrinology and Diabetes, School of Medicine, Faculty of Biology, Medicine and Healthy, University of Manchester, Manchester, UK; 6 Imperial College London, London, UK; 7 Department of Renal Medicine, Manchester University NHS Foundation Trust, Manchester, UK; 8 Bioxydyn Limited, Manchester, UK

**Keywords:** Bioimpedance, Sodium, CKD

## Abstract

Multifrequency bioimpedance spectroscopy (BIS) is an established method for assessing fluid status in chronic kidney disease (CKD). However, the technique is lacking in predictive value and accuracy. BIS algorithms assume constant tissue resistivity, which may vary with changing tissue ionic sodium concentration (Na^+^). This may introduce significant inaccuracies to BIS outputs. To investigate this, we used ^23^Na magnetic resonance imaging (MRI) to measure Na^+^ in muscle and subcutaneous tissues of 10 healthy controls (HC) and 20 patients with CKD 5 (not on dialysis). The extracellular (Re) and intracellular (Ri) resistance, tissue capacitance, extracellular (ECW) and total body water (TBW) were measured using BIS. Tissue water content was assessed using proton density-weighted MRI with fat suppression. BIS-derived volume indices were comparable in the two groups (OH: HC − 0.4 ± 0.9 L vs. CKD 0.5 ± 1.9 L, p = 0.13). However, CKD patients had higher Na^+^ (HC 21.2 ± 3.0, CKD 25.3 ± 7.4 mmol/L; p = 0.04) and significantly lower Re (HC 693 ± 93.6, CKD 609 ± 74.3 Ohms; p = 0.01); Ri and capacitance did not vary. Na^+^ showed a significant inverse linear relationship to Re (r_s_ = − 0.598, p < 0.01) but not Ri. This relationship of Re (y) and Na^+^ (x) is described through equation y = − 7.39x + 814. A 20% increase in tissue ionic Na^+^ is likely to overestimate ECW by 1.2–2.4L. Tissue Na^+^ concentration has a significant inverse linear relationship to Re. BIS algorithms to account for this effect could improve prediction accuracy of bioimpedance derived fluid status in CKD.

**Electronic supplementary material:**

The online version of this article (10.1007/s40620-019-00620-3) contains supplementary material, which is available to authorized users.

## Introduction

Extracellular volume (ECW) expansion is commonly encountered in advanced chronic kidney disease (CKD) [1]. Salt and water retention leading to fluid overload (FO) through loss of renal function presents a significant cardiovascular risk factor and a major clinical challenge, especially in dialysis-dependant CKD with anuria [2–4]. The assessment of FO in CKD through the use of electrical bioimpedance spectroscopy (BIS) has been introduced into clinical practice for more accurate prediction of fluid balance with a potential impact on outcomes [5–7].

BIS utilises the principle that body tissue compartments have different resistance and reactance to different frequencies of alternating electrical current (AC). Low frequencies (≤ 5 kHz) can only transverse the ECW while high frequencies (> 100–200 kHz) propagate through both ECW and intracellular water (ICW) compartments. The work of Thomasset [8, 9] enabled the measurement of the water volume of a compartment with a known length and electrical resistance using the equation [9]:

1 $${\text{V}} = \rho \frac{{L^{2} }}{Z}$$

where V = total body water [TBW (ml)], ρ = tissue resistivity (Ohm cm), L = conductor length (cm), Z = impedance (Ohm).

To measure TBW, BIS assumes that ρ remains constant and the human body behaves as a cylinder with isotropic conductivity [10]. Whole body multifrequency BIS uses multiple frequencies of AC (5–1000 kHz) to measure impedance and phase angle (Φ) at all these frequencies. Impedance is a derivative of tissue resistance and reactance and represents its insulating and conductive ratio [11]. The resistance of a tissue to an electric current is inversely related to its water content while its reactance relates to its cell mass [11], if the assumptions hold true. The relationship between the resistance and reactance is characterised by Φ. This represents the cumulative time delay that occurs when AC transverses through cell membranes [11]. The more cellular the tissue, the higher the Φ. Tissue cellularity and cellular health is also reflected by tissue capacitance (the ability to store electricity for a short period of time). From these measurements the BIS device will derive the extracellular (Re) and intracellular (Ri) resistance and through the use of the Cole–Cole [10] model, and either the Xitron [12] or Moissl [13] equations, estimate ECW and TBW.

The ρ of body fluid compartments is influenced by ions such as sodium (Na^+^), potassium and chloride, and their relative extra- and intracellular distribution [14]. It has been observed that post-dialysis BIS measurements of ICW can markedly vary from pre-dialysis measurements; this has been attributed to electrolyte changes [15]. A key assumption by BIS algorithms is that the ionic tissue composition remains stable in relation to water. However, the variation in tissue electrolyte concentrations and its impact on BIS measurements has not been studied in human subjects.

Na^+^ as the dominant ion in the body is the predominant contributor to tissue ρ. It has been assumed that due to its close affinity to water, high levels of Na^+^ are mirrored by water volume expansion [16, 17]. It has been suggested that Na^+^ could exist in a water-free form, predominantly attached to the proteoglycan matrix of tissues such as the skin [18, 19]. The use of ^23^Na magnetic resonance imaging (MRI) provides a reproducible, non-invasive method for measuring tissue Na^+^ [20]. Studies using this technique have reported high Na^+^ in patients with CKD [21].

In haemodialysis patients BIS has been used to guide fluid ultrafiltration and help adjust the target weight. The highly reproducible nature of BIS (intra- and inter-observer error < 2%) makes this method useful for monitoring fluid status changes in individuals [22]. However, despite a good overall correlation between BIS and isotopic measurements of TBW and ECW [9], the window of error for ECW estimates can be up to 17% [13]. This translates to an intrinsic methodological variation of ± 2.8 L [13] limiting its use in clinical practice. Deviations from euvolaemia within this range have been associated with higher all-cause and cardiovascular mortality in dialysis [7]. Accuracy in ECW prediction therefore may be crucial in improving clinical outcomes in CKD.

We hypothesised that varying tissue Na^+^ concentration can influence impedance measured through BIS and we aimed to assess its impact on BIS predictive accuracy.

## Methods

This is a cross-sectional case–control study examining the association between measurements of Na^+^ tissue concentration derived by ^23^Na MRI and fluid volume measurements from multifrequency BIS in patients with advanced CKD and healthy subjects (HC). In a subgroup of participants, fluid in the lower limb was also assessed using conventional proton MRI. The study received approval by the North West NHS Research Ethics Committee (15/NW/0471). All adult patients with eGFR < 15 ml/min (pre-dialysis) and ability to consent, receiving their care at the participating centre were able to participate in the study. HC were recruited through posters placed at the participating centre. All adults with no history of CKD were eligible. The only exclusion criteria for both groups were contraindication to MRI and limb amputation. Written informed consent was obtained prior to enrolment into the study. All study measurements and blood tests were performed during a single visit by one operator, in a controlled hospital environment. Medical and drug histories were obtained from the participants and their electronic medical records. Participants’ blood samples were analysed for serum electrolytes, urea and creatinine, albumin and osmolality at the participating centre’s NHS laboratory. The laboratory also reported the MDRD eGFR.

### Fluid assessment using BIS

Whole body multifrequency BIS was performed using the Body Composition Monitor (BCM^®^, Fresenius Medical Care, Germany) as per manufacturer's specifications. The device provided measurements of Re, Ri and tissue capacitance, as well as impedance and Φ for individual frequencies (5–1000 kHz). The software, based on impedance, calculated TBW, ECW and ICW.

### MRI

Imaging was performed using a 3T MRI scanner (Philips 3T Achieva). Images were acquired using a dual-tuned ^1^H/^23^Na head coil (RAPID Biomedical GmbH, Germany). Analysis was performed using Horos Image Viewer Version 1.7 (www.horosproject.org).

#### Sodium imaging

**Fig. 1:**
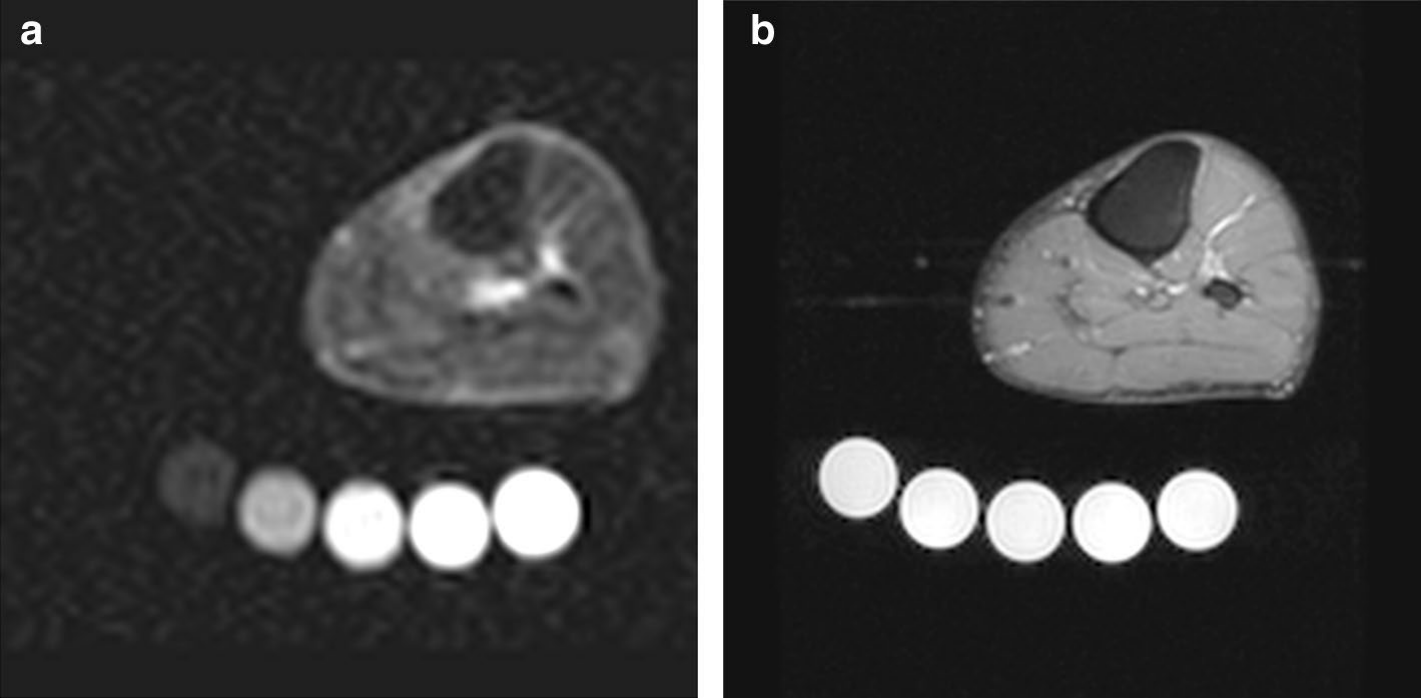
^23^Sodium magnetic resonance image of the left lower limb of a patient with advanced chronic kidney disease (4a). Underneath the calf muscle the five saline phantoms can be seen. ^1^H magnetic resonance image of the left lower limb of a patient with advanced chronic kidney disease acquired using a proton density-weighted scan with SPAIR fat suppression (4b)

An adapted version of the protocol used by Kopp et al. [23] was used for acquiring ^23^Na images between the levels of the left knee and ankle. All images (Fig. 1a) were analysed by two operators and the reported Na ^+^ for muscle (Na_M_^+^) and subcutaneous (Na_SC_^+^) tissue was the average of the two measurements (Supplementary Tables 1 and 2). The average of the Na^+^ concentration of these two tissue compartments was then reported as Na_AveSC+M_^+^ concentration. A detailed method for deriving Na^+^ concentration is presented in Supplementary Material 1. The average method coefficient of variation for signal intensity calibration was 13.9%. The mean inter-reader variation for Na_M_^+^ was 0.07 ± 3.5 mmol/L and Na_SC_^+^ was − 1.21 ± 3.9 mmol/L.

#### MRI tissue water measurement

To analyse tissue water content in the muscle and subcutaneous (SC) tissue, we used an adaptation of the approach used by Dahlmann et al. [21]. ^1^H images were acquired using a proton density-weighted scan with SPAIR fat suppression (Fig. 1b). All images were reported by two operators and the reported muscle and SC tissue fractional water content (FWC) was the average of the two readers. A detailed method for image acquisition and deriving FWC is presented in Supplementary Material 1. The average technique coefficient of variation of signal intensity with the phantom was 2.5%. The mean inter-reader variation of FWC for muscle was 0.019 ± 0.05 and for SC was − 0.027 ± 0.05 (Supplementary Tables 3, 4).

### Statistics

Cohort characteristics and measurements were explored using descriptive epidemiology. Categorical variables were reported as percentages and ratios. Continuous variables were reported as mean (standard deviation) where distribution was normal and as median (minimum and maximum) when distribution was skewed. Normality of distribution was assessed using the Shapiro–Wilk method. Comparison of categorical variables between the study groups was performed using Pearson’s Chi^2^. Group comparison of continuous variables was performed using paired *t* test and Mann–Whitney test based on the distribution.

Associations between BIS measurements, body composition estimations and Na^+^ and water MRI measurements were explored using Pearson’s correlation for normally distributed variables and Spearman’s correlation when variables’ distribution was skewed. Statistical analysis was performed using IBM SPSS Statistics, version 23 (IBM Corp., USA).

## Results

**Fig. 2:**
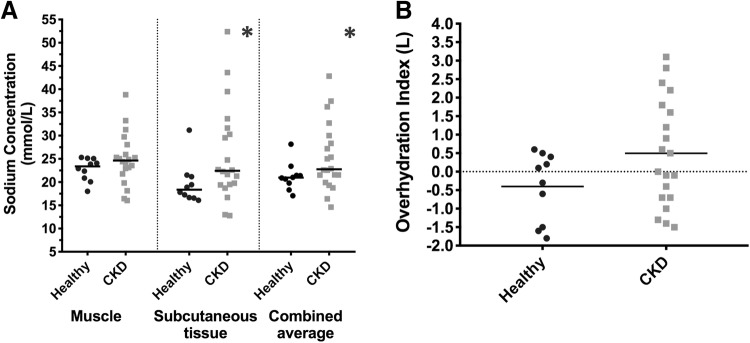
CKD patients have higher total and subcutaneous MR-derived sodium concentration than healthy controls despite comparable volume indices. *CKD* chronic kidney disease, *ECW* extracellular water, *L* litres, *mmol* millimole, *TBW* total body water. *Indicates statistical significance (p < 0.05)

Thirty subjects (10 HC and 20 advanced CKD) were studied. Both cohorts were of similar age (HC: 51.6 ± 13.4 years, CKD: 53.0 ± 9.3 years; p = 0.749) and sex (1:1). The CKD group had higher comorbidity, prevalence of hypertension, pill burden and deranged biochemistry in keeping with advanced CKD (eGFR 11 ± 2.7 ml/min/1.73 m^2^) when compared to HC (86.5 ± 7.4 ml/min/1.73 m^2^) (Supplementary Table 5). Both cohorts had comparable OH (HC − 0.4 ± 0.9L vs. CKD 0.5 ± 1.9L) (Table 1, Fig. 2).

**Table 1:** Participants’ bioimpedance and MR measurements

	Entire cohort	Controls	Advanced CKD	p value
Bioimpedance measurements				
Re (Ohms)	**637.0 (SD 89.2)**	**693.2 (SD 93.6)**	**608.9 (SD 74.3)**	0.012*
Ri (Ohms)	1571.2 (SD 303.3)	1626.9 (SD 338.5)	1543.3 (SD 289.3)	0.486
Capacitance (nF)	1.4 (0.7–3.4)	1.4 (1.1–3.4)	1.4 (0.7–3.2)	0.895
Z (Ohms)				
5 kHz	**611.9 (SD 87.0)**	**664.4 (SD 96.4)**	**585.7 (SD 70.5)**	0.017*
100 kHz	**518.3 (SD 71.1)**	**558.0 (SD 82.6)**	**498.5 (SD 56.9)**	0.028*
Φ (^o^)				
5 kHz	2.5 (1.5–4.4)	2.6 (2.2–4.4)	2.5 (1.5–4.1)	0.311
100 kHz	5.0 (SD 0.8)	5.3 (SD 0.5)	4.9 (SD 0.9)	0.225
Body composition estimations				
TBW/Weight (L/kg)	0.46 (SD 0.08)	0.47 (SD 0.08)	0.46 (SD 0.08)	0.681
ECW	16.3 (SD 2.84)	15.5 (SD 3.30)	16.6 (SD 3.29)	0.314
OH (L)	0.2 (SD 1.3)	− 0.4 (SD 0.9)	0.5 (SD 1.5)	0.087
BMI (kg/m^2^)	27.5 (SD 4.6)	25.7 (SD 4.8)	28.4 (SD 4.4)	0.127
MR-derived Na Conc (mmol/L)				
Na_M_^+^	24.19 (SD 4.8)	22.77 (SD 2.5)	24.90 (SD 5.5)	0.257
Na_SC_^+^	**21.22 (12.79–52.38)**	**18.35 (16.09–31.18)**	**22.43 (12.79–52.38)**	0.031*
Na_AveSC+M_^+^	**23.96 (SD 6.5)**	**21.21 (SD 3.0)**	**25.34 (SD 7.4)**	0.039*
MR-derived water content fraction				
Muscle	0.50 (SD 0.04) (N = 17)	0.49 (SD 0.04) (N = 4)	0.50 (SD 0.05) (N = 13)	0.744
Subcutaneous	**0.24 (SD 0.10) (N = 17)**	**0.18 (SD 0.02) (N = 4)**	**0.26 (SD 0.11) (N = 13)**	0.023*
Serum biochemistry				
Na^+^ (mmol/L)	141.0 (136–144)	140.5 (136–143)	141.5 (137–144)	0.609
Potassium (mmol/L)	4.8 (3.7–5.5)	4.5 (4.0–5.4)	5.0 (3.7–5.5)	0.250
Bicarb (mmol/L)	**21.8 (SD 3.1)**	**24.6 (SD 1.7)**	**20.5 (SD 2.7)**	< 0.001*
Urea (mmol/L)	**16.9 (SD 9.9)**	**4.9 (SD 0.9)**	**23.0 (SD 5.9)**	< 0.001*
Creatinine (mmol/L)	**326.2 (SD 208.8)**	**70.2 (SD 10.4)**	**454.2 (SD 121.4)**	< 0.001*
eGFR (ml/min/1.73 m^2^)	**36.2 (SD 36.5)**	**86.5 (SD 7.4)**	**11.0 (SD 2.7)**	< 0.001*
Albumin (g/L)	38 (29–42)	39 (35–42)	37 (29–40)	0.051
Osmolality (mOsm/kg)	**301.5 (274–338)**	**290.5 (274–298)**	**307.0 (293–338)**	< 0.001*

The bold value is used for correlation co-efficient and p-values with statistical significance

*Bicarb* bicarbonate, *BMI* body mass index, *CKD* chronic kidney disease, *Conc* concentration, *ECW* extracellular water, *eGFR* estimated glomerular filtration rate, *g* gram, *kg* kilogram, *L* litre, *m* metre, *min* minute, *ml* millilitre, *mmol* millimole, *mOsm* millliosmole, *MR* magnetic resonance, *Na*^*+*^ sodium, *Na*_*AveSC+M*_^*+*^ average of muscle and subcutaneous sodium, *Na*_*M*_^*+*^ muscle sodium, *Na*_*SC*_^*+*^ subcutaneous sodium, *nF* nanofarad, *OH* overhydration index, *Re* extracellular resistance, *Ri* intracellular resistance, *SD* standard deviation, *TBW* total body water, *Z* impedance, *Φ* phase angle

*Indicates significance of < 0.05

### Tissue Na^+^ concentration

CKD participants had significantly higher Na_AveSC+M_^+^ (HC: 21.2 ± 3.0 mmol/L, CKD: 25.3 ± 7.4 mmol/L; p = 0.039) and Na_sc_^+^(HC: 19.7 ± 4.5 mmol/L, CKD: 25.8 ± 10.3 mmol/L; p = 0.031) (Table 2, Fig. 2). Variations in Na_M_^+^ were also observed, but were not significantly different between the cohorts. Measurement data are provided in Supplementary Tables 2, 3. OH correlated significantly with Na_AveSC+M_^+^ (Table 2).

**Table 2:** Correlation of bioimpedance measurements and bioimpedance-derived body composition to MR-derived tissue sodium concentration for the entire cohort

	Na_M_^+^		Na_SC_^+^		Na_AveSC+M_^+^	
	r	p value	r	p value	r	p value
Serum osmolality	0.222	0.239	**0.488**	0.006*	**0.382**	0.037*
Bioimpedance measurements						
Re	**− 0.415**	0.023*	**− 0.694**	< 0.001*	**− 0.598**	< 0.001*
Ri	0.160	0.397	**−** 0.257	0.170	**−** 0.050	0.795
Capacitance	**− 0.402**	0.028*	0.010	0.956	**−** 0.206	0.276
Z						
5 kHz	− **0.423**	0.020*	− **0.712**	< 0.001*	− **0.609**	< 0.001*
100 kHz	**−** 0.289	0.122	**− 0.650**	< 0.001*	**− 0.499**	0.005*
Φ						
5 kHz	**− 0.481**	0.007*	**−** 0.169	0.372	**− 0.372**	0.043*
100 kHz	**− 0.634**	< 0.001*	**− 0.454**	0.012*	**− 0.588**	0.001*
Body composition estimations						
TBW/weight	**−** 0.128	0.501	0.145	0.444	**−** 0.017	0.928
ECW	0.217	0.249	**0.507**	0.004*	**0.384**	0.036*
OH	**0.495**	0.005*	**0.432**	0.017*	**0.507**	0.004*

The bold value is used for correlation co-efficient and p-values with statistical significance

The relationship between variables was assessed using Spearman’s correlation. *ECW* extracellular water, *Na*_*AveSC+M*_^*+*^ average of muscle and subcutaneous sodium, *Na*_*M*_^*+*^ muscle sodium, *Na*_*SC*_^*+*^ subcutaneous sodium, *OH* overhydration index, *r* correlation coefficient, *Re* extracellular resistance, *Ri* intracellular resistance, *TBW* total body water, *Z* impedance, *Φ* phase angle

*Indicates significance of < 0.05

**Table 3:** Correlation of multifrequency bioimpedance measurements, bioimpedance**—**derived body composition and MR-derived tissue sodium concentration to tissue fractional water content measured by MRI for the entire cohort

	Muscle water fraction		Subcutaneous water fraction	
	r	p value	r	p value
Bioimpedance measurements				
Re	**−** 0.362^^^	0.145	**−** 0.419	0.094
Ri	**−** 0.475^^^	0.054	**−** 0147	0.573
Capacitance	**−** 0.223	0.390	**−** 0.287	0.264
Z				
5 kHz	**−** 0.377^^^	0.135	**−** 0.441	0.076
100 kHz	**−** 0.433^^^	0.075	**−** 0.343	0.178
Φ				
5 kHz	0.174^^^	0.503	**−** 0.161	0.538
100 kHz	0.086^^^	0.742	**−** 0.424	0.090
Body composition estimations				
TBW/weight	0.418^^^	0.095	0.252	0.328
ECW	0.396^^^	0.232	0.164	0.529
OH	0.006^^^	0.980	0.258	0.318
Na_M_^+^ Conc	**−** 0.086	0.743	**0.502**	0.040*
Na_SC_^+^ Conc	0.172	0.510	**0.726**	**< 0.001**
Na_AveSC+M_^+^	0.044	0.866	**0.681**	0.003*

The bold value is used for correlation co-efficient and p-values with statistical significance

The relationship between variables was assessed using Pearson’s correlation for normally distributed variables and Spearman’s correlation when distribution was skewed. *ECW* extracellular water, *kg* kilogram, *L* litre, *MRI* magnetic resonance imaging, *Na*_*AveSC+M*_^*+*^ average of muscle and subcutaneous sodium, *Na*_*M*_^*+*^ muscle sodium, *Na*_*SC*_^*+*^ subcutaneous sodium, *OH* overhydration index, *r* correlation coefficient, *Re* extracellular resistance, *Ri* intracellular resistance, *TBW* total body water, *Z* impedance, *Φ* phase angle

^^^Denotes Pearson’s correlation coefficient

*Indicates significance of < 0.05

### Tissue impedance in CKD

Impedance at both low (5 kHz) and high (100 kHz) frequencies was significantly lower in the CKD group resulting in higher volume estimates (Table 1). Re was also significantly lower in CKD (HC: 693 ± 94 Ohms, CKD: 609 ± 74 Ohms; p = 0.012) but Ri, capacitance and Φ did not vary between the two groups (Table 1).

### Tissue Na^+^, impedance and BIS-derived body composition

**Fig. 3:**
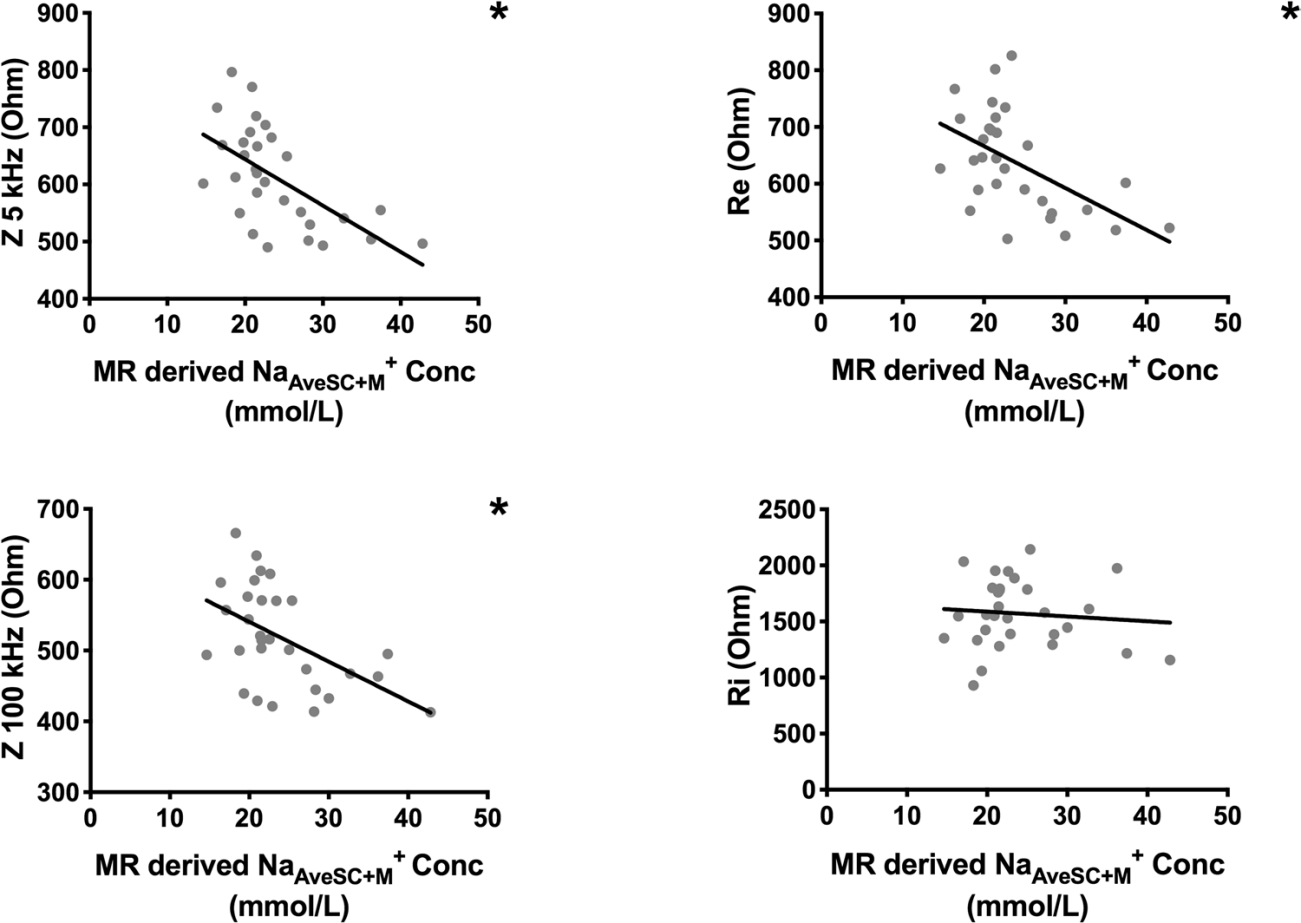
Correlation of sodium concentration to tissue impedance, extracellular and intracellular resistance. The graphs demonstrate that MR-derived sodium concentration correlates to whole body impedance at both low and high electrical current frequencies as well as extracellular resistance. *Indicates statistical significance (*p* < 0.05). *Conc* concentration, *kg* kilogram, *kHz* kilohertz, *L* litre, *mmol* millimole, *MR* magnetic resonance, *Na*^*+*^ sodium, *Re* extracellular resistance, *Ri* intracellular resistance, *Z* impedance

Re exhibited an inverse relationship with tissue Na^+^ concentrations for both muscle and skin compartments (Na_M_^+^: r = − 0.415, p = 0.023; Na_SC_^+^: r = − 0.694, p < 0.001; Na_AveSC+M_^+^: r = − 0.598, p < 0.001) (Table 2, Fig. 3). The relationship is described by the linear equation

2 $${\text{Re}} = - 7.39Na_{AveSC + M}^{ + } + 814$$

Higher Na_M_^+^ was also associated with low Φ at low current frequencies and capacitance (Table 2). Na_SC_^+^ and Na_AveSC+M_^+^ correlated negatively with impedance measurements at all frequencies (Table 3).

### Tissue FWC, its distribution and association to Na^+^

**Fig. 4:**
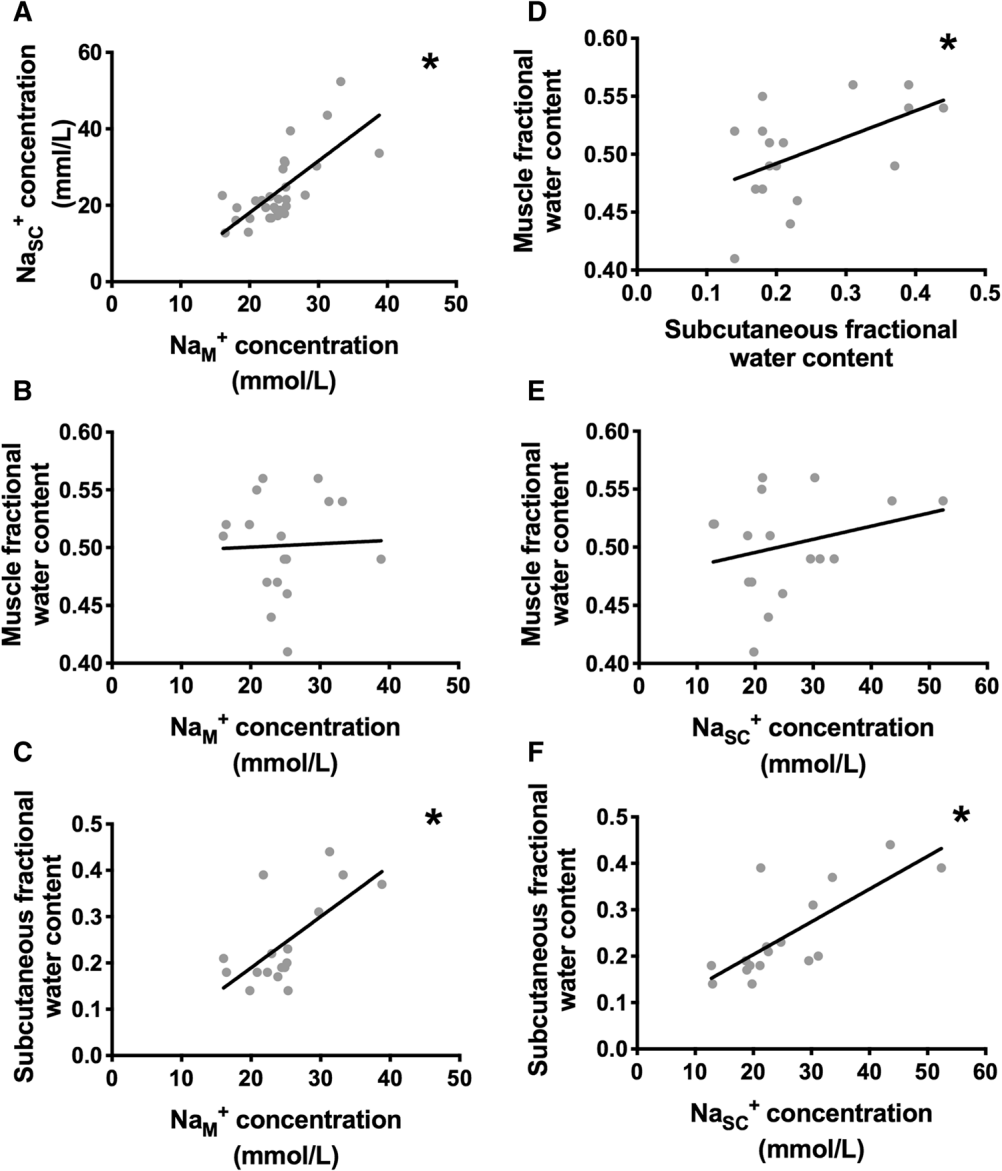
Correlations between compartmental MR-derived sodium concentration and tissue fractional water content. Pictures A and D demonstrate that both MR-derived Na^+^ concentration and fractional content in the subcutaneous tissue correlate to that imaged in the muscle. Picture C and F demonstrate that subcutaneous fractional water but not muscle water content (Pictures B and E) correlate to both muscle and subcutaneous sodium concentration. *Indicates statistical significance (p < 0.05). *Conc* concentration, *L* litre, *mmol* millimole, *Na*^*+*^ sodium

Seventeen participants (4 HC, 13 CKD) completed imaging to the same area of their lower limb for estimation of the FWC in muscle and SC. FWC did not correlate with tissue resistivity indices or BIS-derived volume parameters. However, FWC in SC correlated significantly with tissue Na^+^ concentration (Table 3, Fig. 4).

### The influence of tissue Na^+^ on Re and ECW estimations

The values within 2 SD from the mean Na_AveSC+M_^+^ concentration in healthy controls were used as the normal reference range for this analysis (15.2–27.2 mmol/L). The equivalent Re reference range (701.8–613.1Ohms) was derived from Eq. 2. In the CKD cohort, the maximum and minimum Na_AveSC+M_^+^ measurements were 42.8 and 14.6 mmol/L, which represented a deviation of − 3.9 to 57.4% (net 60%) from the normal reference range. Considering that 5% variation in Re could result in a 2.5% variation in ECW [24], a 20% change in Re can translate to a 1.2–2.4 L ECW discrepancy.

## Discussion

To our knowledge, this is the first study in humans to investigate the relationship between tissue Na^+^ concentration derived using MRI and body impedance. Our results indicate that Na^+^ concentrations correlate with impedance at both low and high frequencies, and overall Re. This means that high Na^+^ concentrations were also linked to BIS-derived ECW readings.

The observed phenomenon of Re dependence to Na^+^ has been previously demonstrated by Rees et al. in their experimental rat model [14]. When an intravenous infusion of hypertonic saline was used to increase body Na^+^ concentration this resulted in a disproportionate rise of BIS-estimated ECW relative to true water volume expansion measured using tracer dilution methods (4–6% vs. 0.5%). Similar to our findings, this was due to an increase in Re. Ri was not influenced by tissue Na^+^ in either of the studies. This is an important limitation of BIS algorithms and could lead to inaccuracies in characterising fluid volume states for clinical management.

Overall changes to Re can lead to overestimation of ECW and Ward et al. have demonstrated that an increase in Re of 10% can lead to a 4.9% change in ECW [24]. Changes in plasma Na^+^ concentration during haemodialysis of up to 3.5% have been linked to changes in Re of 3.2% [25] so it is conceivable that variations in tissue Na^+^ and osmolality can lead to ECW overestimation. Based on our findings, a 10% increase in tissue Na^+^ concentration can lead to an overestimation of ECW by up to 1.2L. Re adjustment to increasing tissue resistivity would improve BIS accuracy and aid clinical decision making, with more accurate target weight prediction in dialysis [22].

Factors that lead to the observed differences in tissue Na^+^ concentration are not fully understood. We attempted to examine this through MR tissue FWC assessment and examine its relationship to tissue Na^+^. In a series of human and animal experimental models Kopp et al. have demonstrated that tissue Na^+^ concentration measured through ^23^Na MRI, although lower than the Na^+^ concentration of the same tissue measured through chemical analysis and ashing, exhibits a very close correlation [23]. This close association was true for both muscle and SC [23]. We demonstrated similar values of MRI-derived Na^+^ concentration to those seen by Kopp et al. [26]. Both groups exhibited similar Na_M_^+^ concentration but increased Na_SC_^+^ concentration in CKD cohort. The observed differences in muscle and skin could be due to different MRI relaxation properties of the two tissues. For this reason, we also chose to analyse the tissue compartments separately in addition to the combined Na_AveSC+M_^+^ measure.

At higher frequencies, although Na_SC_^+^ was also linked to lower impedance, Na_M_^+^ was more closely associated with low tissue capacitance, which may indicate reduced body cellularity. The replacement of muscle mass with fat tissue and ECW is a phenomenon observed with aging and in CKD [27]. Previous studies have shown Na_SC_^+^ accumulation independent of water accumulation [26]. Our study showed a good correlation between tissue Na^+^ and SC FWC. Our findings do not contradict the existence of osmotically-inactive Na^+^ but suggest that in states of deranged salt and water homeostasis the MR-imaged Na^+^ concentration may not be entirely independent of water content. Whether part of the imaged Na^+^ also detected the osmotically-inactive ions is unclear and cannot be determined by the techniques used in this study.

The methods used in our study provide a non-invasive approach for measuring water and Na^+^ content, but there are certain intrinsic limitations. Our estimates of FWC are derived from MRI signals related to those obtained from pure water. While our acquisition protocol minimised the influence of other MRI parameters (chiefly relaxation times), we cannot discount the possibility that some influence from such confounders may remain. Although our study population size is comparable to similar studies using MRI for Na^+^ imaging, the subgroup of participants that underwent measurement of FWC was relatively small and presented a limitation to our analysis. Both MRI methods used are unable to differentiate between the extracellular and intracellular compartments in the imaged tissue. In fact, MRI as well as BIS assume uniformity of extracellular to intracellular tissue across different parts of the body. This assumption has to be considered when interpreting our findings. Although BIS has been validated in a number of studies in health and CKD [9, 13, 28, 29], there are a number of limitations [15, 30] and one such has been demonstrated in this study.

In summary, the study demonstrates an inverse and predictable relationship between Re and varying tissue Na^+^ concentration. The attributable discrepancy in Re estimations could lead to significant over- or underestimation of ECW volumes. Higher precision in BIS-guided fluid volume measurements could be achieved with adjustments to algorithms to account for this effect.

## Electronic supplementary material

Below is the link to the electronic supplementary material.

Supplementary material 1 (DOCX 119 kb)
