# Supplementary material for: Extracellular resistance is sensitive to tissue sodium status; implications for bioimpedance-derived fluid volume parameters in chronic kidney disease
Source: J Nephrol. 2019 Jun 18;33(1):119–27. doi: 10.1007/s40620-019-00620-3 (PMC7007413; doi:10.1007/s40620-019-00620-3)
Supplement: Supplementary file 1 — Supplementary material 1 (DOCX 119 kb) [file 40620_2019_620_MOESM1_ESM.docx]

## Supplementary Material

**Supplementary material 1**

**MR Sodium Image analysis**

To derive the TNC from the MRI signal intensity, five saline calibration phantoms, each with varying Na concentrations (15, 45, 80, 115 and 150 mmol/L), were used (Figure 1a). Regions of interest (ROI) were drawn over the saline phantoms for each scan at three different slice levels. The averages of signal intensity for each phantom were plotted against the respective Na concentrations. The slope and y-intercept of the line of best fit were used to calculate the Na concentration from tissue signal intensity. Three ROIs were then drawn in the muscle and three in the subcutaneous (SC) tissue, at different slice levels for each scan, and the average Na concentration for each compartment was calculated. All images were analysed by two operators and the reported Na for muscle (Na^M^) and SC (Na^SC^) is the average of the two measurements (Supplementary Tables 1 and 2). The average of the Na concentration of these two tissue compartments was then reported as Na^AveSC+M^ concentration. The average method coefficient of variation for signal intensity calibration was 13.9%. The mean inter-reader variation for Na^M^ was 0.07±3.5mmol/L and Na^SC^ was -1.21±3.9mmol/L.

**MR Image analysis to measure tissue water**

The signal intensity for water was taken to equal that of the 15mmol/L saline phantom. Muscle and SC tissue water content was measured as the signal intensity of an ROI drawn in each of these compartments expressed as a fraction of the signal intensity from the phantom at the same slice level. This was measured at three different levels and the average tissue fractional water content (FWC) was derived. All images were reported by two operators and the reported muscle and SC tissue FWC is the average of the two readers. The average technique coefficient of variation of signal intensity with the phantom was 2.5%. The mean inter-reader variation of FWC for muscle was 0.019±0.05 and for SC was -0.027±0.05 (Supplementary Tables 3-4).

**Supplementary Table 1 MRI-derived muscle sodium concentrations**

| **Muscle Sodium Concentration (mmol/L)** | | | | | | | | | |
| --- | --- | --- | --- | --- | --- | --- | --- | --- | --- |
| **Participants** | **Reader 1** | | | | **Reader 2** | | | | **Average of R1 and R2** |
|  | **ROI 1** | **ROI 2** | **ROI 3** | **Average** | **ROI 1** | **ROI 2** | **ROI 3** | **Average** |  |
| **Controls** |  |  |  |  |  |  |  |  |  |
| **C1** | 18.99 | 27.26 | 26.07 | **24.11** | 27.23 | 23.99 | 26.88 | **26.03** | **25.07** |
| **C2** | 17.95 | 17.02 | 20.92 | **18.63** | 18.56 | 19.35 | 14.30 | **17.41** | **18.02** |
| **C3** | 24.98 | 24.41 | 25.88 | **25.09** | 29.99 | 24.96 | 21.61 | **25.52** | **25.31** |
| **C4** | 24.76 | 24.55 | 23.88 | **24.40** | 26.09 | 24.07 | 21.09 | **23.75** | **24.07** |
| **C5** | 18.53 | 17.24 | 20.52 | **18.76** | 24.97 | 21.08 | 18.13 | **21.39** | **20.05** |
| **C6** | 22.38 | 23.67 | 27.03 | **24.36** | 22.64 | 22.44 | 19.37 | **21.49** | **22.92** |
| **C7** | 24.28 | 24.11 | 28.53 | **25.64** | 18.28 | 28.36 | 27.27 | **24.64** | **25.14** |
| **C8** | 19.47 | 26.80 | 25.72 | **24.00** | 24.35 | 24.60 | 22.25 | **23.73** | **23.86** |
| **C9** | 22.03 | 22.41 | 22.70 | **22.38** | 19.62 | 16.16 | 22.32 | **19.36** | **20.87** |
| **C10** | 22.03 | 23.06 | 23.58 | **22.89** | 21.25 | 22.21 | 22.04 | **21.83** | **22.36** |
| **CKD** |  |  |  |  |  |  |  |  |  |
| **CKD1** | 24.52 | 22.54 | 30.07 | **25.71** | 22.56 | 24.19 | 21.12 | **22.63** | **24.17** |
| **CKD2** | 23.03 | 24.73 | 22.58 | **23.45** | 26.16 | 30.34 | 23.25 | **26.61** | **25.03** |
| **CKD3** | 24.33 | 23.65 | 26.12 | **24.70** | 28.44 | 24.88 | 28.15 | **27.16** | **25.93** |
| **CKD4** | 25.57 | 26.01 | 23.59 | **25.06** | 20.88 | 21.20 | 21.57 | **21.22** | **23.14** |
| **CKD5** | 27.22 | 30.16 | 25.57 | **27.65** | 30.64 | 28.66 | 26.15 | **28.48** | **28.07** |
| **CKD6** | 18.31 | 28.50 | 23.78 | **23.56** | 28.37 | 16.08 | 22.55 | **22.33** | **22.95** |
| **CKD7** | 23.17 | 30.92 | 26.25 | **26.78** | 24.37 | 24.00 | 22.84 | **23.73** | **25.26** |
| **CKD8** | 29.47 | 31.25 | 32.25 | **30.99** | 29.73 | 28.74 | 26.92 | **28.47** | **30.41** |
| **CKD9** | 13.71 | 15.71 | 18.97 | **16.13** | 15.85 | 15.27 | 19.18 | **16.76** | **13.19** |
| **CKD10** | 35.76 | 31.23 | 33.71 | **33.57** | 33.70 | 60.55 | 37.96 | **44.07** | **38.82** |
| **CKD11** | 33.79 | 36.24 | 33.23 | **34.41** | 31.30 | 34.26 | 30.58 | **32.05** | **33.23** |
| **CKD12** | 22.71 | 24.57 | 25.71 | **24.24** | 25.84 | 23.27 | 27.26 | **25.46** | **24.85** |
| **CKD13** | 25.23 | 26.36 | 26.17 | **25.92** | 20.63 | 25.41 | 22.67 | **22.90** | **24.41** |
| **CKD14** | 21.45 | 18.95 | 19.58 | **19.99** | 22.52 | 24.85 | 23.18 | **23.52** | **24.41** |
| **CKD15** | 14.45 | 15.65 | 19.24 | **16.45** | 14.99 | 14.88 | 17.05 | **15.64** | **16.04** |
| **CKD16** | 23.53 | 31.09 | 27.87 | **27.50** | 20.08 | 17.18 | 21.67 | **19.65** | **23.57** |
| **CKD17** | 14.59 | 16.52 | 16.28 | **15.80** | 20.35 | 19.75 | 21.40 | **20.50** | **18.15** |
| **CKD18** | 22.85 | 25.20 | 23.97 | **24.05** | 28.66 | 24.75 | 26.39 | **26.60** | **25.32** |
| **CKD19** | 19.76 | 16.76 | 21.97 | **19.49** | 22.01 | 14.86 | 23.54 | **20.14** | **19.82** |
| **CKD20** | 32.43 | 24.21 | 29.82 | **28.73** | 37.13 | 33.67 | 30.39 | **33.73** | **31.28** |

The table contains muscle sodium concentration in mmol/L for each participant as derived by the 2 scan readers. CKD= chronic kidney disease, L= Litre, mmol= millimole, R= reader, ROI= region of interest.

**Supplementary Table 2. MRI-derived subcutaneous tissue sodium concentrations**

| **Subcutaneous Sodium Concentration (mmol/L)** | | | | | | | | | |
| --- | --- | --- | --- | --- | --- | --- | --- | --- | --- |
|  | **Reader 1** | | | | **Reader 2** | | | | **Average of R1 and R2** |
|  | **ROI 1** | **ROI 2** | **ROI 3** | **Average** | **ROI 1** | **ROI 2** | **ROI 3** | **Average** |  |
| **Control** |  |  |  |  |  |  |  |  |  |
| **C1** | 18.66 | 18.02 | 18.23 | **18.30** | 17.87 | 17.57 | 16.55 | **17.33** | **17.82** |
| **C2** | 13.77 | 12.82 | 20.14 | **15.57** | 16.42 | 14.30 | 19.13 | **16.62** | **16.09** |
| **C3** | 23.43 | 26.99 | 24.37 | **24.93** | 19.37 | 17.95 | 17.00 | **18.10** | **21.52** |
| **C4** | 17.49 | 13.35 | 17.41 | **16.08** | 15.79 | 24.28 | 15.16 | **18.41** | **17.25** |
| **C5** | 16.48 | 16.60 | 14.59 | **15.89** | 19.19 | 16.75 | 15.72 | **17.22** | **16.55** |
| **C6** | 17.92 | 13.45 | 22.38 | **17.92** | 16.31 | 14.98 | 14.93 | **15.41** | **16.66** |
| **C7** | 29.32 | 34.15 | 25.63 | **29.70** | 35.88 | 32.97 | 29.16 | **32.67** | **31.18** |
| **C8** | 26.38 | 14.89 | 17.01 | **19.43** | 16.12 | 18.15 | 20.81 | **18.36** | **18.89** |
| **C9** | 22.10 | 25.52 | 23.82 | **23.81** | 16.21 | 15.53 | 23.77 | **18.50** | **21.16** |
| **C10** | 19.83 | 23.06 | 19.21 | **20.70** | 18.85 | 16.88 | 18.72 | **18.15** | **19.43** |
| **CKD** |  |  |  |  |  |  |  |  |  |
| **CK1** | 23.52 | 27.22 | 20.74 | **23.83** | 19.08 | 21.12 | 18.09 | **19.43** | **21.63** |
| **CKD2** | 31.29 | 31.31 | 26.17 | **29.59** | 22.69 | 36.42 | 42.05 | **33.72** | **31.65** |
| **CKD3** | 44.02 | 46.68 | 47.07 | **45.93** | 36.74 | 33.67 | 28.76 | **33.06** | **39.49** |
| **CKD4** | 15.20 | 21.46 | 15.67 | **17.44** | 15.15 | 17.68 | 14.91 | **15.91** | **16.68** |
| **CKD5** | 22.58 | 26.66 | 21.08 | **23.44** | 23.54 | 21.74 | 20.47 | **21.92** | **22.68** |
| **CKD6** | 31.47 | 19.20 | 20.03 | **23.57** | 28.29 | 16.55 | 18.17 | **21.00** | **22.28** |
| **CKD7** | 28.63 | 30.18 | 22.51 | **27.11** | 22.35 | 21.29 | 23.60 | **22.41** | **24.76** |
| **CKD8** | 33.90 | 24.85 | 32.47 | **30.41** | 28.84 | 26.98 | 35.66 | **30.16** | **30.28** |
| **CKD9** | 11.50 | 14.23 | 13.85 | **13.19** | 9.64 | 10.80 | 16.72 | **12.39** | **12.79** |
| **CKD10** | 35.72 | 32.17 | 31.73 | **33.21** | 30.53 | 40.07 | 31.55 | **34.05** | **33.63** |
| **CKD11** | 61.32 | 43.72 | 50.21 | **51.75** | 58.37 | 53.02 | 39.28 | **50.22** | **52.38** |
| **CKD12** | 26.69 | 24.01 | 27.43 | **26.07** | 28.05 | 31.14 | 39.85 | **33.01** | **29.54** |
| **CKd13** | 18.86 | 14.93 | 19.07 | **17.61** | 25.10 | 15.78 | 18.51 | **19.80** | **18.71** |
| **CKD14** | 22.10 | 17.50 | 22.83 | **20.81** | 20.24 | 22.41 | 22.62 | **21.76** | **21.28** |
| **CKD15** | 22.07 | 25.42 | 21.86 | **23.12** | 23.00 | 21.86 | 21.25 | **22.04** | **22.58** |
| **CKD16** | 20.08 | 21.82 | 29.59 | **23.83** | 14.71 | 14.72 | 15.96 | **15.13** | **19.48** |
| **CKD17** | 21.68 | 17.75 | 20.73 | **20.05** | 20.50 | 21.40 | 14.32 | **18.74** | **19.39** |
| **CKD18** | 19.48 | 19.72 | 20.51 | **19.90** | 17.13 | 20.87 | 20.91 | **19.64** | **19.77** |
| **CKD19** | 13.89 | 13.41 | 13.13 | **13.48** | 11.36 | 13.45 | 12.51 | **12.50** | **12.99** |
| **CKD20** | 46.14 | 43.79 | 40.66 | **43.53** | 40.88 | 49.48 | 40.56 | **43.64** | **43.59** |

The table contains subcutaneous tissue sodium concentration in mmol/L for each participant as derived by the 2 scan readers. CKD= chronic kidney disease, L= Litre, mmol= millimole, R= reader, ROI= region of interest.

**Supplementary Table 3. MRI-derived muscle fractional water content.**

| **Muscle Water Fraction** | | | | | | | | | |
| --- | --- | --- | --- | --- | --- | --- | --- | --- | --- |
| **Participants** | **Reader 1** | | | | **Reader 2** | | | | **Average of R1 and R2** |
|  | **ROI 1** | **ROI 2** | **ROI 3** | **Average** | **ROI 1** | **ROI 2** | **ROI 3** | **Average** |  |
| **Controls** |  |  |  |  |  |  |  |  |  |
| **C7** | 0.45 | 0.48 | 0.48 | **0.47** | 0.49 | 0.48 | 0.55 | **0.51** | **0.49** |
| **C8** | 0.41 | 0.45 | 0.35 | **0.40** | 0.51 | 0.52 | 0.58 | **0.53** | **0.47** |
| **C9** | 0.58 | 0.56 | 0.48 | **0.54** | 0.58 | 0.55 | 0.54 | **0.57** | **0.55** |
| **C10** | 0.49 | 0.49 | 0.45 | **0.48** | 0.51 | 0.45 | 0.45 | **0.47** | **0.47** |
| **CKD** |  |  |  |  |  |  |  |  |  |
| **CKD6** | 0.43 | 0.45 | 0.42 | **0.43** | 0.44 | 0.44 | 0.43 | **0.44** | **0.44** |
| **CKD7** | 0.52 | 0.43 | 0.44 | **0.46** | 0.46 | 0.39 | 0.50 | **0.45** | **0.46** |
| **CKD8** | 0.55 | 0.53 | 0.59 | **0.56** | 0.55 | 0.59 | 0.58 | **0.57** | **0.56** |
| **CKD9** | 0.55 | 0.60 | 0.46 | **0.54** | 0.54 | 0.53 | 0.44 | **0.50** | **0.52** |
| **CKD10** | 0.50 | 0.46 | 0.50 | **0.49** | 0.49 | 0.50 | 0.51 | **0.50** | **0.49** |
| **CKD11** | 0.64 | 0.53 | 0.43 | **0.53** | 0.52 | 0.56 | 0.55 | **0.54** | **0.54** |
| **CKD12** | 0.49 | 0.42 | 0.48 | **0.46** | 0.52 | 0.49 | 0.56 | **0.52** | **0.49** |
| **CKD13** | 0.60 | 0.52 | 0.49 | **0.54** | 0.48 | 0.47 | 0.49 | **0.48** | **0.51** |
| **CKD14** | 0.58 | 0.53 | 0.56 | **0.56** | 0.64 | 0.54 | 0.48 | **0.55** | **0.56** |
| **CKD15** | 0.47 | 0.46 | 0.44 | **0.46** | 0.60 | 0.62 | 0.48 | **0.57** | **0.51** |
| **CKD18** | 0.32 | 0.47 | 0.44 | **0.41** | 0.42 | 0.38 | 0.40 | **0.40** | **0.41** |
| **CKD19** | 0.54 | 0.47 | 0.45 | **0.49** | 0.53 | 0.55 | 0.57 | **0.55** | **0.52** |
| **CKD20** | 0.58 | 0.51 | 0.55 | **0.55** | 0.55 | 0.54 | 0.53 | **0.54** | **0.54** |

The table contains subcutaneous tissue fractional water content for each participant as derived by the 2 scan readers. CKD= chronic kidney disease, R= reader, ROI= region of interest.

**Supplementary Table 4. MRI-derived subcutaneous tissue fractional water content.**

| **Subcutaneous Water Fraction** | | | | | | | | | |
| --- | --- | --- | --- | --- | --- | --- | --- | --- | --- |
|  | **Reader 1** | | | | **Reader 2** | | | | **Average of R1 and R2** |
|  | **ROI 1** | **ROI 2** | **ROI 3** | **Average** | **ROI 1** | **ROI 2** | **ROI 3** | **Average** |  |
| **Control** |  |  |  |  |  |  |  |  |  |
| **C7** | 0.24 | 0.23 | 0.20 | **0.22** | 0.19 | 0.19 | 0.17 | **0.18** | **0.20** |
| **C8** | 0.16 | 0.14 | 0.15 | **0.15** | 0.16 | 0.19 | 0.19 | **0.18** | **0.17** |
| **C9** | 0.21 | 0.19 | 0.20 | **0.20** | 0.15 | 0.15 | 0.17 | **0.20** | **0.18** |
| **C10** | 0.19 | 0.16 | 0.19 | **0.18** | 0.19 | 0.18 | 0.19 | **0.19** | **0.18** |
| **CKD** |  |  |  |  |  |  |  |  |  |
| **CKD6** | 0.22 | 0.23 | 0.26 | **0.24** | 0.20 | 0.19 | 0.19 | **0.20** | **0.22** |
| **CKD7** | 0.29 | 0.26 | 0.25 | **0.27** | 0.19 | 0.17 | 0.22 | **0.19** | **0.23** |
| **CKD8** | 0.39 | 0.34 | 0.27 | **0.33** | 0.27 | 0.27 | 0.29 | **0.28** | **0.31** |
| **CKD9** | 0.21 | 0.21 | 0.14 | **0.18** | 0.20 | 0.16 | 0.19 | **0.18** | **0.18** |
| **CKD10** | 0.42 | 0.44 | 0.34 | **0.40** | 0.33 | 0.37 | 0.34 | **0.35** | **0.37** |
| **CKD11** | 0.47 | 0.46 | 0.34 | **0.42** | 0.30 | 0.43 | 0.33 | **0.35** | **0.39** |
| **CKD12** | 0.15 | 0.17 | 0.21 | **0.18** | 0.12 | 0.23 | 0.23 | **0.20** | **0.19** |
| **CKd13** | 0.18 | 0.18 | 0.17 | **0.18** | 0.20 | 0.19 | 0,21 | **0.20** | **0.19** |
| **CKD14** | 0.44 | 0.44 | 0.47 | **0.45** | 0.40 | 0.39 | 0.22 | **0.36** | **0.39** |
| **CKD15** | 0.20 | 0.20 | 0.19 | **0.20** | 0.24 | 0.23 | 0.18 | **0.22** | **0.21** |
| **CKD18** | 0.14 | 0.14 | 0.18 | **0.15** | 0.15 | 0.12 | 0.11 | **0.13** | **0.14** |
| **CKD19** | 0.12 | 0.12 | 0.13 | **0.12** | 0.15 | 0.15 | 12.51 | **0.15** | **0.14** |
| **CKD20** | 0.53 | 0.50 | 0.44 | **0.49** | 0.35 | 0.43 | 0.41 | **0.40** | **0.44** |

The table contains subcutaneous tissue fractional water content for each participant as derived by the 2 scan readers. CKD= chronic kidney disease, R= reader, ROI= region of interest.

**Supplementary Table 5. Participants’ demographic characteristics**

|  | | **Entire Cohort (n=30)** | **Controls (n=10)** | **Advanced CKD (n=20)** | **p-value** |
| --- | --- | --- | --- | --- | --- |
| **Age** | | 52.5 (SD 10.6) | 51.6 (SD 13.4) | 53.0 (SD 9.3) | 0.749 |
| **Sex: Male** | | 15 (50%) | 5 (50%) | 10 (50%) | 1.000 |
| **Ethnicity** | **White** | 27 (90.0%) | 9 (90.0%) | 18 (90.0%) | 1.000 |
|  | **Black** | 3 (10.0 %) | 1 (10.0%) | 2 (10.0%) |  |
| **Charlson Comorbidity Index** | | **2.5 (0-6)** | **1 (0-3)** | **3 (2-6)** | **<0.001*** |
| **Diabetes mellitus** | | 4 (13.3%) | 1 (10.0%) | 3 (15.0%) | 0.704 |
| **Hypertension** | | **23 (76.7%)** | **3 (30.0%)** | **20 (100%)** | **<0.001*** |
| **Cardiovascular disease** | | 1 (3.3 %) | - | 1 (5.0%) | 0.427 |
| **Smoking** | | 5 (16.7 %) | 2 (20.0%) | 3 (15.0%) | 0.729 |
| **N^o^ of medication** | | **6.0 (0-17)** | **1.0 (0-4)** | **6.5 (3-17)** | **<0.001*** |
| **N^o^ of BP medication** | | **2 (0-6)** | **0 (0-2)** | **2 (0-6)** | **<0.001*** |
| **Diuretic treatment** | | 5 (16.7%) | - | 5 (25.0%) | 0.083 |
| **Primary cause of renal disease** | **APKD** |  |  | 2 (10%)  3 (15%)  1 (5%)  2 (10%)  1 (5%)  3 (15%)  5 (25%)  3 (15%) |  |
|  | **Glomerulonephritis** |  |  |  |  |
|  | **Renovascular/Ischemic/HTN** |  |  |  |  |
|  | **Diabetes** |  |  |  |  |
|  | **Obstructive uropahty** |  |  |  |  |
|  | **Reflux nephropathy** |  |  |  |  |
|  | **Uncertain aetiology** |  |  |  |  |
|  | **Other** |  |  |  |  |
| **Failing transplant** | |  |  | 5 (25%) |  |
| **Previous dialysis treatment** | |  |  | 5 (25%) |  |
| **Cumulative dialysis vintage (months)** | |  |  | 35.6 (SD 32.9) |  |

APKD= adult polycystic kidney disease, BP= blood pressure, CKD= chronic kidney disease, HTN= hypertension, SD= standard deviation. * indicates significance of <0.05.
